# Purification of Arabinoxylans from Corn Fiber and Preparation of Bioactive Films for Food Packaging

**DOI:** 10.3390/membranes10050095

**Published:** 2020-05-11

**Authors:** Maria Serra, Verónica Weng, Isabel M. Coelhoso, Vitor D. Alves, Carla Brazinha

**Affiliations:** 1 LEAF—Linking Landscape, Environment, Agriculture and Food, Instituto Superior de Agronomia, Universidade de Lisboa, Tapada da Ajuda, 1349-017 Lisboa, Portugal; mariamanuelserra94@gmail.com (M.S.); vitoralves@isa.ulisboa.pt (V.D.A.); 2 LAQV-REQUIMTE, Chemistry Department, Faculdade de Ciências e Tecnologia, Universidade Nova de Lisboa, 2829-516 Caparica, Portugal; v.weng@campus.fct.unl.pt (V.W.); imrc@fct.unl.pt (I.M.C.)

**Keywords:** corn fiber, arabinoxylans, polysaccharides, ultrafiltration, diafiltration, bioactive films

## Abstract

Corn fiber, a by-product of the starch industry, is presently incorporated in animal feed. However, it has arabinoxylans as added-value components (besides ferulic acid) that should be valorized. In this work, the raw material, a fraction enriched in arabinoxylans from corn fiber, previously produced by alkaline extraction from corn fiber and pre-concentrated by ultrafiltration, was further purified. The use of ultrafiltration operated in diafiltration mode (dia-ultrafiltration) was evaluated for the purification of the arabinoxylans fraction. The objective was to maximize the removal of the small contaminants from the fraction and to maximize the permeability and/or the permeate flux, by selecting the relevant operating conditions involved in this process. The removal of contaminants (%) was estimated when their apparent rejection stabilized. Edible films were produced, from the resultant purified arabinoxylans fraction, using glycerol as plasticizer (30% dry basis). Additionally, films with the incorporation of ferulic acid were developed, in order to obtain barriers with antioxidant activity. The films were characterized in terms of mechanical properties, antioxidant activity and permeability to water vapor. The films prepared presented a good potential to be used as packaging for food products with low water content.

## 1. Introduction

Petroleum-based polymers (plastics) have dominated the packaging market in the past years, in various industries, due to their favorable properties (mechanical performance, stability, impermeability and ease of sterilization along with simple, low-cost production), representing 99% of all polymeric materials on the global market [1,2]. Conventional plastics include polyethylene (PE), polyethylene terephthalate (PET), polystyrene (PS), polypropylene (PP), polyvinyl chloride (PVC) and others [2]. The production of plastics uses non-renewable feedstocks and releases great amounts of greenhouse gases. Plastics’ resistance to degradation makes their presence persistent in the environment and their burning releases many toxic gases due to the additives present in the plastic composition [1]. Therefore, the constant increase in demand for packaging materials raises an environmental concern, since most of these materials are difficult to reuse and recycle [2].

This led to a search for alternative materials, such as biopolymers. Biopolymers are gaining great importance from an environmental point of view, due to their great advantage of being biodegradable, and from an economical point of view, as their production follows a biorefinery approach of the valorization of low cost and renewable feedstocks [2]. The global biopolymers market was 2465.4 million USD in 2017 and is estimated to be 7196.8 million USD by 2024 [3] and the World Edible Packaging (in which Polysaccharides are included) was 697 million USD in 2016 and is expected to increase 1.57 times [4].

Among biopolymers extracted from nature, an important class is the polysaccharides, which is very abundant and diverse. Polysaccharides applications in food and medicine are huge [5]. Some biopolymers in this category include chitin, chitosan (both with animal origins), starch (plant origin), cellulose (plant and bacterial origins), pectins and alginates [1,2,5]. Polysaccharide materials for packaging and coating applications, with selective barrier and/or bioactive properties, have been reported such as pectin, starch, chitosan, xylan, lignin and cellulose [2,6]. As examples, pectin films were prepared with the addition of plasticizers such as glycerol and choline chloride to improve their properties [7]. Yam starch films integrated with eugenol were prepared for pork preservation using d-sorbitol as plasticizer [8]. The biodegradable microbial polysaccharide Fucopol was used to produce films that were studied as packaging material for walnuts [9]. In addition to passive barriers, active films, namely with antioxidant activity, have also been developed, such as chitosan films enriched with olive pomace [10] and gelatine films with the incorporation of papaya peel microparticles [11]. Polysaccharides exhibit excellent gas barrier properties (against oxygen and carbon dioxide), but low resistance to water (due to their hydrophilic nature), low mechanical performance and (still) a high price [2]. To overcome these drawbacks, the packaging development using less studied polysaccharides and blends of polysaccharides with other biodegradable polymers should be addressed.

Similar to cellulose, hemicellulose is very abundant in nature. Both are present in plants cells walls, together they create a structure that provides rigidity to the plant. The main backbone can be substituted with different groups, making their composition very diverse [12]. Among hemicellulose polysaccharides, xylan is the most predominant type. This polysaccharide is constituted by a backbone of d-xylopyranose units linked by a β-(1-4) linkage. When these units have arabinose units as side groups, the structure is denominated arabinoxylan. Arabinoxylan can be found in cereal grains in significant amounts [12,13]. Corn fiber, a by-product of the corn wet-milling, a step of the corn processing industry, is presently used in animal feed applications. However, it is composed of added-value components that should be valorized, such as arabinoxylans and ferulic acid. The arabinoxylans from corn bran, a by-product of corn dry-milling (similar to corn fiber) [14], was reported to have a molecular weight of 253/362 kDa [15], 300–500 kDa [16] or 264.2 ± 9.72 kDa (when extracted at 25 °C) [17].

The purification of polysaccharides is challenging, as their structure is highly complex and they are in complex media [18]. The reported purification methods are precipitation, chromatography methods, which always involved the addition of compounds, ultracentrifugation and preparative zone electrophoresis [19].

An integrated process of microfiltration and ultrafiltration units (used in the purification of polysaccharides of *Sinorhizobium meliloti*) [20], or solely an ultrafiltration process, are easy operating processes to concentrate the polysaccharides and to remove to a certain extent the small compounds. Nevertheless, the process might be time-consuming due to the high viscosity of polysaccharide solutions and membrane fouling [18]. An alternative to avoid membrane fouling is dialysis, a process operating under very mild operating conditions. Dialysis was carried out to purify the arabinoxylan from maize bran, where the small contaminants were removed through the membrane by diffusion at 23 °C for 3 days [21].

In an ultrafiltration process operated in a diafiltration mode (dia-ultrafiltration), the volume of the feed solution is constant, as the permeate flowrate is the same as the flowrate of the solvent (water). Therefore, dia-ultrafiltration has the advantages of enhancing the removal of the small contaminants, when compared to ultrafiltration, and preventing the viscosity of the feed solution to rise over time. Dia-ultrafiltration under controlled transmembrane pressure conditions was used in the purification of pectin from mature citrus peel after being concentrated by crossflow microfiltration [22], in the purification of high molecular weight polysaccharides from white wine [23] and in the purification of Fucopol, an exopolysaccharide from *Enterobacter* A47, using a hollow fiber membrane Model UFP-500-E-6A, GE Healthcare) with a Molecular Weight Cut-Off of 500 kDa operating at a transmembrane pressure of 0.7 bar [24].

Arabinoxylans were also applied in the production of biodegradable films, such as those from wheat bran, maize bran or dried distillers grain using glycerol or sorbitol as a plasticizer [25]. Furthermore, films from arabinoxylan extracted from sorghum bran, bagasse and biomass were also prepared using glycerol as plasticizer [26].

In the present work, dia-ultrafiltration operated under controlled permeate flux conditions was examined as the technology to purify polysaccharides, in this case, a pre-treated arabinoxylans fraction from corn fiber (after a previous alkaline extraction, a rough purification by ultrafiltration and a dilution to turn the fraction processable by membranes). This technology operates under mild conditions (milder than when operated under controlled transmembrane pressure conditions), adding no other compounds than water, and it is more productive than dialysis. The relevant operating conditions were selected: temperature, relaxation procedure and Reynolds number at the feed compartment. The impact of the different operating conditions on the performance of purification was quantitatively evaluated by using different parameters (1—percentage of loss of hydraulic permeability during the purification experiment, 2—the observed transmembrane pressure conditions and 3—the rejection and the removal from the feed compartment of the charged contaminants, in sodium chloride equivalents, and of the contaminants, in ferulic acid equivalents). The novelty of this work relies on the detailed study on polysaccharides purification by dia-ultrafiltration under controlled transmembrane pressure conditions. This work aimed at proposing this technology (involving neither organic solvents nor other additives, besides water) to be used on the purification of polysaccharides, in general, and arabinoxylans, in particular.

Additionally, the potential of purified arabinoxylans by dia-filtration to produce biodegradable films was studied. The films produced were characterized in terms of morphology, color, mechanical properties and permeability to water vapor. In addition, films with antioxidant activity were developed with the incorporation of ferulic acid. The use of this compound as an active agent is a sustainable approach, as it is one of the compounds present in arabinoxylan extracts.

## 2. Materials and Methods

### 2.1. Materials

An extract from corn fiber was produced in the research group of the LAQV-REQUIMTE, FCT NOVA through an alkaline extraction, which was further processed by membranes in the concentration mode of operation. The resultant concentrated fraction, concentrated 5×, enriched in the large compounds arabinoxylans (arabinoxylans fraction), was the raw material in this work. Therefore, this work addresses the purification of a part of an arabinoxylan extract (obtained by alkaline extraction), but the study of the recovery of arabinoxylan from corn fiber by alkaline extraction is out of the scope of this paper. After dilution of the raw material with water (to turn it processable by membranes), its purification was carried out using a hollow fiber membrane module operating inside-out with an area of 0.48 m^2^ (GE Healthcare, Chicago, IL, USA; Model: UFP-100-C-6A) [27]. It was composed of 520 polysulfone fibers, with an internal diameter of 0.5 mm, a length of 0.60 m and a molecular weight cut-off (MWCO) of 100 Da. The maximum temperature and maximum feed pressure (at the values of temperature between 25 to 50 °C) recommended by the producer were 80 °C and 3.4 bar, respectively. The hydraulic permeability measured with deionized water at 23 °C before the experiments was 224.7 ± 19.09 L·h^−1^·m^−2^·bar^−1^. The membrane unit was operated at controlled permeate flux conditions.

The selected value of MWCO was 100 kDa, a value of MWCO below the sizes of corn arabinoxylans reported in literature (between 253/362 kDa (604) [15], 300–500 kDa [16]), to assure the rejection of the arabinoxylans. Ultrasil 110 and Ultrasil 75 from Ecolab (Mississauga, ON Canada), ethanol (97%) from Panreac (Barcelona, Spain) and potassium metabisulfite (≥98%) from Sigma-Aldrich (St. Louis, MI, USA) were used in membrane cleaning procedures and conservation. Glycerol (99%) was purchased from Fisher Chemical (Loughborough, UK) and ferulic acid (99%) from Sigma-Aldrich (St. Louis, MI, USA) to incorporate in the films. Magnesium nitrate (≥98%), magnesium chloride (≥98%) and ferric chloride solution (≥99.99%) from Sigma-Aldrich (St. Louis, MI, USA) and TPTZ solution (≥98.5%) from Thermo Fisher Scientific (Loughborough, UK) were used in the analyses for film characterization.

### 2.2. Purification of the Arabinoxylans Extracts: Membrane Procedure

Each experiment had duplicate measurements and the values presented were calculated with the average value of the duplicates and the deviation was calculated with the standard deviation of the duplicates.

#### 2.2.1. Modes of Operation and Experimental Set-Ups

**Figure 1:**
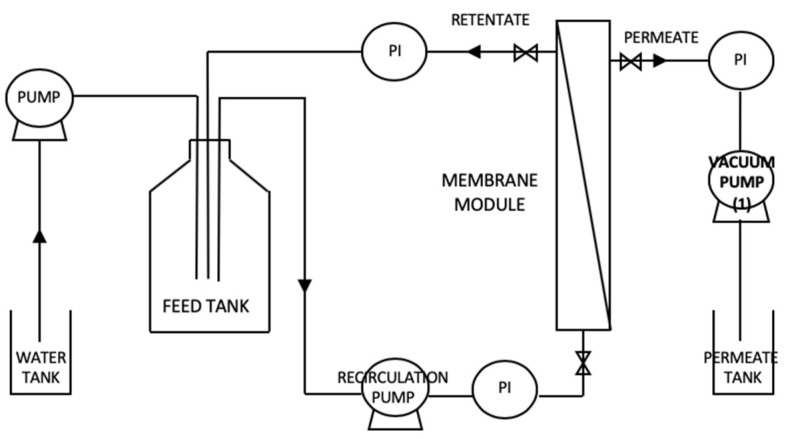
Representation of the membrane unit, operated in the dia-ultrafiltration mode, used in this work: a hollow fiber unit, operated under controlled permeate flux pressure conditions, which is controlled by the permeate vacuum pump (1). Legend: PI, pressure gauge.

The experiments for the purification of the arabinoxylans were operated in the diafiltration mode (dia-ultrafiltration experiments, as ultrafiltration membranes were used) and the diafiltration volume *D* (-) is an important parameter, which was calculated with Equation (1):

(1) $$D=\frac{V_{w}}{V_{feed}}$$

where *V_w_* (L) is the volume of the solvent (water) added and *V_feed_* (L) is the volume of the feed compartment. The experiments of purification of the (diluted) raw material were performed in the hollow fiber membrane unit, of which the experimental set-up is detailed in Figure 1. The unit operates under controlled permeate flux conditions (similarly to the operation of Membrane Bioreactors MBR), where the permeate flux is controlled by the frequency value of a pump in the permeate side of the membrane, related to the permeate pressure. The pressure at the feed stream is slightly above the atmospheric pressure and the permeate stream is slightly below the atmospheric pressure. This mode of operation represents a milder way to operate than the controlled transmembrane pressure operation, and is applied when low membrane fouling conditions are intended.

Membrane cleaning was done in three stages to recover the initial hydraulic permeability: an alkaline solution with a concentration of 0.5% (*w*/*w*) of Ultrasil 110 recirculated for 30 min. Then, an acid solution of Ultrasil 75 with a concentration of 0.03% (*w*/*w*) recirculated for 20 min. The last step consisted of circulating ethanol for 20 min with a concentration of 70% (*w*/*w*). Between the different passages, the membrane was washed with deionized water until the pH stabilized at 7 and until a negligible conductivity was reached. To keep the operation characteristics of the membrane and to prevent microbiological exposure the membrane was preserved in a solution containing 1% of potassium metabisulfite.

During the dia-ultrafiltration experiments, the permeate was collected in a vessel and its mass was measured with an electronic balance. The volumetric permeate flux, the permeability, the apparent rejection factor of contaminants *i* and the percentage of the removal of the contaminants *i* were calculated. The volumetric permeate flux *J_perm_* (L·m^−2^·h^−1^) is calculated by Equation (2):

(2) $$J_{perm}=\frac{m_{perm}}{\rho \;.A\;.t}$$

where *m_perm_* (Kg) is the mass of permeate, ρ (Kg/m^3^) is the density of the permeate (1000 kg/m^3^ for water), *A* (m^2^) is the membrane area and *t* (h) is the time of permeation. The permeability, *Lp* (L·m^−2^·h^−1^·bar^−1^), is the permeate flux divided by the transmembrane pressure *TMP* (bar). The apparent rejections of (mostly small) contaminants, $R_{i}$(%) and the percentage of the removal of the (mostly small) contaminants *i* were calculated during the experiments through Equations (3) and (4), respectively:

(3) $$R_{i}=1-\frac{C_{i,perm}}{C_{i,feed}}$$

(4) $$\%\;Removal\left(compound_{i}\right)=1-\frac{C_{i,feed\;}\left(t\right)}{C_{i,feed}\;\left(t=0\right)},\;i=\;\mathrm{contaminants}$$

where $c_{i,perm}$ (g/L) is the permeate concentration (instantaneous, non-cumulative) of small contaminants in the permeate, $c_{i,feed}$ (g/L) is the retentate concentration of contaminants in the feed compartment (retentate) referred to the same time of $c_{i,perm}$ (g/L) and wherein Equation (4), $c_{i,feed}$(*t*) (g/L) and $c_{i,feed}$(*t* = 0) (g/L) are the concentrations of small contaminants in the feed compartment at a certain time of experiment *t* and at the initial time of the experiment respectively. The type of contaminants considered in this work and how their concentration values are obtained is explained in Section 2.3.

#### 2.2.2. Operating Conditions

The optimization of the purification of the (diluted) raw material was carried out by assessing the relevant operating conditions: temperature, relaxation procedure and Reynolds number at the feed compartment *Re_feed_* (-) (related to the feed hydrodynamic conditions). The dia-ultrafiltration experiments are summarized in Table 1. 0.6 L of the (diluted) raw material was processed in each experiment. In the experiments for the optimization of the temperature and the relaxation procedure, the final diafiltration volume was 35. Considering the results of these experiments, on the experiments for the optimization of the *Re_feed_* (-), the final diafiltration volume was decided to be shortened to 10 (see Section 3.2.3).

### 2.3. Characterization of the Raw Material and the Fractions Produced by Dia-Ultrafiltration

Each sample was analyzed in terms of charged contaminants and of contaminants. Each analysis had duplicate measurements and the values presented were calculated with the average value of duplicates and the deviation was calculated with the standard deviation of the duplicates.

#### 2.3.1. Viscosity of the Raw Material at Different Dilution Ratios

The raw material (see Section 2.1) of this work was very viscous due to the presence of arabinoxylans; therefore, the raw material had to be diluted to reach a viscosity close to the viscosity of water, to turn the extract processable by membranes. The apparent viscosity of diluted raw material at different dilution ratios was measured by loading directly the samples on a cone and plate geometry (diameter 35 mm, angle 2°) of a controlled stress rheometer (HAAKE MARSIII, Thermo Fisher Scientific), coupled with a Peltier system for temperature control measured at 20 °C and 40 °C. Flow curves were obtained using a steady-state flow ramp in the shear rate range from 0.1 to 1000 s^−1^.

#### 2.3.2. Quantification of Charged Contaminants

The concentration of the contaminants in the (diluted) raw material and in the corresponding fractions obtained from dia-ultrafiltration processing (see Section 2.1) were quantified by the conductivity (related to the charged compounds present) model sension+ EC71 GLP (HACH, Loveland, CO, USA) and expressed in the concentration of sodium chloride equivalents, (g_NaCl_/L). The calibration curve was obtained using different concentrations of sodium chloride (30, 15, 7.5, 3.75, 1.88, 9.38 × 10^−1^, 4.69 × 10^−1^, 2.34 × 10^−1^, 1.17 × 10^−1^, 5.86 × 10^−2^, 2.93 × 10^−2^, 1.46 × 10^−2^, 7.32 × 10^−3^, 3.66 × 10^−3^, 1.83 × 10^−3^, 9.16 × 10^−4^, 4.58 × 10^−4^, 2.29 × 10^−4^, 1.14 × 10^−4^ g/L).

#### 2.3.3. Quantification of Contaminants

The concentration of the small contaminants in the (diluted) raw material and in the corresponding fractions obtained from dia-ultrafiltration processing was also quantified by absorbance at 280 nm wavelength (related to proteins and phenolic compounds) with a UV–Vis spectrophotometer (model: Evolution 21, Thermo Scientific, USA) and expressed in the concentration of ferulic acid equivalents, g_FA_/L. The calibration curve was obtained using different concentrations of ferulic acid (1, 5 × 10^−1^, 2.5 × 10^−1^, 1.3 × 10^−1^, 6.3 × 10^−2^, 3.1 × 10^−2^, 1.6 × 10^−2^, 8 × 10^−3^, 4 × 10^−3^, 2 × 10^−3^, 1 × 10^−3^ g/L).

### 2.4. Preparation and Characterization of Films

#### 2.4.1. Films Preparation

The film preparation method was adapted from Anderson & Simsek, (2019) [21]. The purified solution, with an initial soluble solids content of 0.2% (*w*/*w* solution), was concentrated to a final solids content of 1.3% (*w*/*w* solution) in a Buchi Rotavapor R-200 system (Marshall scientific, Hampton, NH, USA), operated under a controlled pressure of 17 mbar and a bath temperature of 40 °C. This solution had a content of 6% (*w*/*w* dry basis) of ferulic acid equivalents. Afterward, the concentrated AX solution was stirred on a hot plate (Velp Scientifica, Usmate, Italy) at 90 °C for 15 min, followed by the addition of glycerol (30% *w*/*w*, dry basis). Ferulic acid was also added at this point up to a final concentration of 10% (*w*/*w* dry basis) of ferulic acid equivalents, but only for producing films enriched in this compound. Then, the film solutions were heated again under stirring for 10 min at 90 °C. Afterward, a mass of 30 g of each solution was transferred to a 55 mm diameter polystyrene Petri dish (Frilabo, Portugal), and dried at 60 °C for 15 h using a thermal oven (BINDER, Tuttlingen, Germany). After, films were peeled from the Petri dishes and were stored for 15 h in a desiccator containing a saturated solution of magnesium nitrate with a relative humidity of 52.9%, before their characterization.

#### 2.4.2. Films Characterization

##### Thickness Measurement

The thickness of the films was measured in duplicate with a digital micrometer (Digimatic Micrometer, Mitutoyo, Japan).

##### Color Measurement

The measurement of the color of the films was carried out in triplicate using a colorimeter (Chroma Meter CR-400, Konica Minolta, Japan), using the CIELAB system. The colorimeter was calibrated with a white standard (L* = 94.62; a* = -0.53 and b* = 3.64, where L* indicates lightness and a* and b* are chromaticity coordinates: a* from red to green and b* from blue to yellow). The hue (°) was calculated according to the following Equation (5):

(5) $$h{}^{\circ}=\arctan\left(\frac{b^{*\;}}{a^{*}}\right)*180/\pi ,\;\mathrm{for}\;a*>\;0\;\mathrm{and}\;b*>\;0$$

Measurements were carried out against the white standard.

##### Films Morphology

Films surface morphology was observed by scanning electron microscopy (SEM) (Jeol JSM-7001F scanning electron microscope, Akishima, Tokyo, Japan) with an operating voltage of 20 kv. Samples were coated with Au/Pd before analysis. The films were observed at a magnification of 5000×.

##### Water Vapor Permeability

The films’ water vapor permeability (WVP) was determined gravimetrically based on the method used by Alves, Costa, & Coelhoso, 2010 [28]. Duplicates of the films after stabilization (see Section 2.4) were cut into 60 mm diameter samples, and sealed at the top of cylindrical glass cups with a solution of magnesium chloride (a_w_ = 0.328). This assembly was placed in a desiccator with a saturated salt solution magnesium nitrate (a_w_ = 0.529), equipped with a fan to promote air circulation inside the desiccator and minimize resistance to mass transfer above the film. The conditions of relative humidity outside the cups and ambient temperature were monitored using a thermohygrometer (Hanna Instruments, Woonsocket, RI, USA). The cups with the films were weighted every 2 h during 12 h to measure the water vapor flux.

##### Tensile Tests

Tensile tests were performed at room temperature (22 °C) using a TA-XT plus texture analyzer (Stable Micro Systems, Surrey, UK) equipped with a 5 kg load cell. Films were cut in rectangular samples (25 mm × 60 mm), fixed with tensile grips, and extended until rupture at a constant speed of 1 mm.s^−1^. The tensile stress at break was calculated as the ratio of the maximum force to the films’ initial cross-sectional area and the strain at break was determined as the ratio of the extension of the sample upon rupture by the initial gage length. The samples’ Young modulus was determined as the slope of the linear initial section of the stress-strain curve. Three film replicates were analyzed.

#### 2.4.3. Antioxidant Activity by Ferric Reduction Antioxidant Power (FRAP)

A solution of FRAP reagent was prepared with 25 mL of 0.3 M acetate buffer, 2.5 mL of 10 mM TPTZ solution and 2.5 mL of ferric chloride solution. Then, 270 µL of nanopure water, 60 μL of methanol, 2.7 mL of FRAP reagent solution and a film sample (1.73 ± 0.23 mg) were added to a test tube. The mixture was homogenized in a vortex (RSLAB, LaborSpirit Lda, Loures, Portugal) and left to incubate for 30 min, at 37 °C in a shaking bath (Thermo scientific, USA) in the absence of light. In the end, the absorbance was measured at λ = 515 nm A standard curve was obtained by using Trolox standard solution at various concentrations (ranging from 100 μM to 2000 μM). The antioxidant activity was expressed as Trolox Equivalent antioxidant Activity (TEAC), as μmol Trolox.mg^−1^ film. The triplicates of the samples were analyzed.

### 2.5. Statistical Analysis

A statistical analysis was performed using the software Statistica v7.0.6.1.0 EM. Tukey’s post-hoc tests with a significance level of 0.05 were used to compare the arabinoxylan films in terms of color, mechanical properties and water vapor permeability.

## 3. Results and Discussion

### 3.1. Viscosity of the Raw Material at Different Dilution Ratios

**Figure 2:**
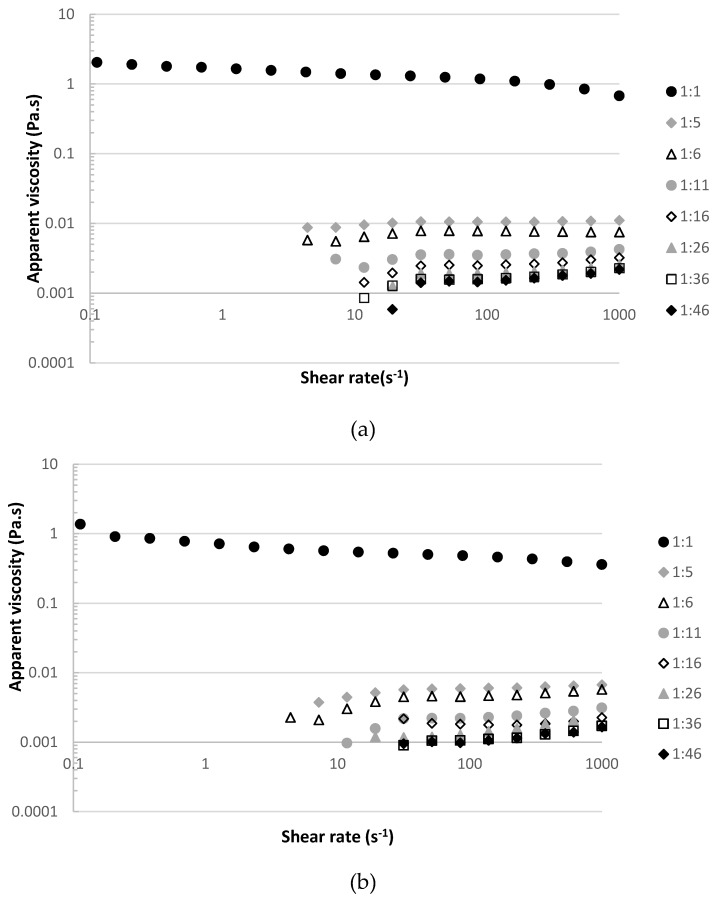
Apparent viscosity (Pa·s) against the shear rate (s^−1^) of the raw material arabinoxylans extract diluted at different dilution ratios: (**a**) at 20 °C and (**b**) at 40 °C.

In order to find the optimum dilution ratio of the raw material (the arabinoxylan fraction, see Section 2.1) corresponding to a viscosity close to the water viscosity at 20 °C, 1 × 10^−3^ Pa·s, with the minimum dilution rate possible (to turn the raw material processable by membranes), the apparent viscosity of the raw material at different ratios of dilution were measured at 20 °C and 40 °C. Figure 2 plots the apparent viscosity of each diluted raw material against the shear rate at 20 °C and 40 °C.

The selected dilution rate of the raw material was 1:16, as the values of apparent viscosity at this dilution ratio were around 2.0 × 10^−3^ Pa·s at 20 °C and 1.8 × 10^−3^ Pa·s at 40 °C.

### 3.2. Optimization of the Purification of the (Diluted) Raw Material by Dia-Ultrafiltration

The purpose of this work is to prove that dia-ultrafiltration, operated under permeate flux conditions, is a reliable technology to purify arabinoxylans from the (diluted) raw material. More specifically, this work evaluated the effect of each relevant operating condition on the purification performance quantified by 1—percentage of loss of hydraulic permeability during the purification experiment (desirably low), 2—the observed transmembrane pressure, which are related to membrane fouling phenomena, 3—the average permeability (desirably high) and 4—the rejection and the removal from the feed compartment of the small contaminants, in ferulic acid equivalents and sodium chloride NaCl equivalents (desirably low and high, respectively).

#### 3.2.1. Effect of Temperature

The impact of the temperature on the purification of the (diluted) raw material was evaluated by performing purification experiments at 30, 40 and 50 °C, under controlled conditions of permeate flux *J_perm_* of 18.8 L·h^−1^·m^−2^, without any relaxation procedure. Table 2 shows the temperature, the hydraulic permeability loss, the transmembrane pressure, the Reynolds number at the feed compartment and the average permeability measured for each temperature.

**Figure 3:**
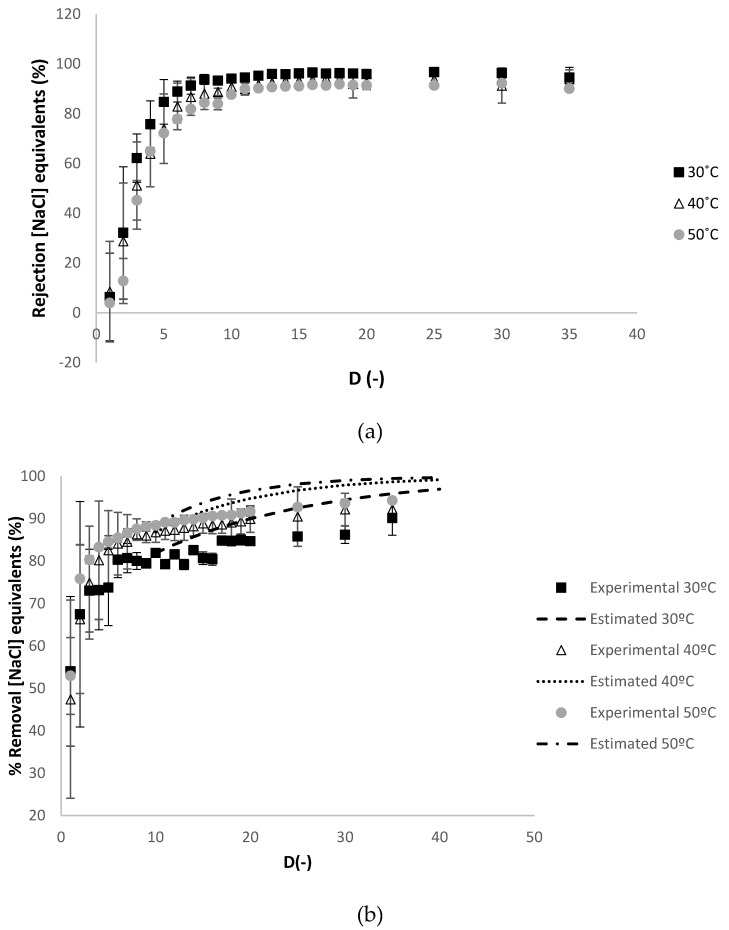
Dia-ultrafiltration experiment at 30 °C, 40 °C and 50 °C with (diluted) arabinoxylans fraction as feed, without relaxation procedure: (**a**) $R_{NaCl}$ (% NaCl.eq), apparent rejection in sodium chloride equivalents and (**b**) percentage of removal of charged contaminants in sodium chloride equivalents (NaCl.eq) against the *D* (-) diafiltration volume. Each analysis had duplicate measurements.

As shown in Table 2, the hydraulic permeability loss was relatively high but similar in these experiments. At 50 °C, the Reynolds number at the feed compartment had the highest value, as expected, as it had the lowest viscosity, which contributed to better feed hydrodynamic conditions and, hence, better mass transport through the membrane. Additionally, at 50 °C, in a process operated under controlled permeate flux conditions, a slightly lower transmembrane pressure value is related to lower membrane fouling phenomena, which, along with a higher value of Re(feed), led to a slightly higher average permeability. Figure 3 presents the progression of the apparent rejection factor (%), Figure 3a and the percentage of removal of the charged contaminants in NaCl equivalents against the diafiltration volume, Figure 3b.

As shown in Figure 3a, the apparent rejections of the charged contaminants were low at low values of diafiltration volume, corresponding to the rejection of the small contaminants that easily permeates. At increasing values of diafiltration volume, the apparent rejection increased until a plateau was reached at values near 100%, corresponding most probably to the big size charged contaminants that remained in the retentate and the smaller charged contaminants that did not permeate due to some membrane fouling. This trend of apparent rejection of contaminants against the diafiltration volume was observed in each purification experiment performed in this work regarding charged contaminants (in NaCl equivalents, NaCl.eq) and contaminants (in Ferulic acid equivalents, FA.eq) present in the raw material. In Figure 3b, in the range of diafiltration volume values where the apparent rejection remained constant, the percentage of removal of charged contaminants (in. NaCl.eq) and contaminants (in FA.eq) may be estimated by Equation (6), explained in detail in the Supplementary Materials, based on [29]:

(6) $$c_{i,feed}\left(t\right)=c_{i,feed}\left(t_{1}\right)\cdot exp\left[-\left(D-D_{1}\right)\cdot \left(1-R_{i}\right)\right]$$

where *t*_1_ (h) and *D*_1_ (-) are, respectively, the values of time of permeation and diafiltration volume when the apparent rejection of the contaminants *i*, *R_i_* (%), becomes constant, until the end of the experiment.

Equation (6) is also applied in each purification experiment performed in this work. In Figure 3b, the percentage of removal of the charged contaminants was estimated at 30 °C, 40 °C and 50 °C, at diafiltration volumes higher than 10, calculated with the experimental apparent rejection of charged contaminants at diafiltration volume of 10 (94%, 91% and 88%, respectively), which is considered constant in this range of diafiltration volumes (see Figure 3a).

**Figure 4:**
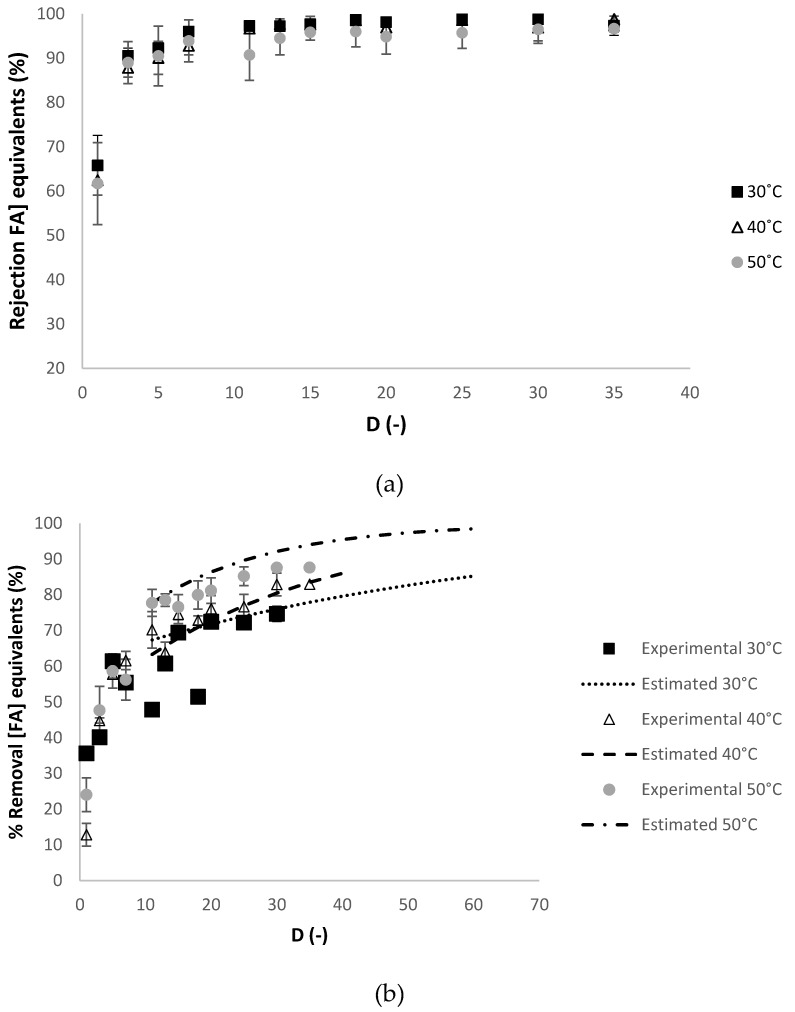
Dia-ultrafiltration experiment at 30 °C, 40 °C and 50 °C with a (diluted) arabinoxylans fraction as feed, without membrane relaxation procedure: (**a**) $R_{FA}$ (% FA.eq), apparent rejection for contaminants equivalents and (**b**) percentage of removal of charged contaminants in ferulic acid equivalents (FA.eq) against the *D* (-) diafiltration volume. Each analysis had duplicate measurements.

A very good agreement between the experimental and the estimated values of the percentage of removal of the charged contaminants in sodium chloride equivalents is observed at 30 °C. Figure 4 presents the evolution of the apparent rejection factor (%) and the percentage of removal of the contaminants in ferulic acid equivalents against the diafiltration volumes.

In Figure 4b, the percentage of removal of the contaminants was estimated at 30 °C, 40 °C and 50 °C, at diafiltration volumes higher than 10, calculated with the experimental apparent rejection of contaminants at diafiltration volume of 10 (98%, 97% and 95%, respectively), which is considered constant in this range of diafiltration volumes (see Figure 4a).

A very good agreement between the experimental and the estimated values of the percentage of removal of the contaminants in ferulic acid equivalents is observed at 30 °C and 50 °C. For the charged contaminants (in NaCl.eq), the apparent rejection and the percentage of removal were similar at 40 °C and 50 °C, considering the associated errors of the measurements (see Figure 3a,b). For the contaminants (in FA.eq), the values of apparent rejection of contaminants were all very similar at all temperatures studied (see Figure 4a). While the percentage of removal of contaminants (in FA.eq) were similar at 40 °C and 50 °C, considering the associated errors of the measurements (see Figure 4b). As the experiments at 40 °C and at 50 °C performed similarly, the experiment at 40 °C was selected, as it consumes less energy than at 50 °C.

#### 3.2.2. Effect of the Relaxation Procedure

The impact of the relaxation procedure on the purification of the (diluted) raw material was evaluated by comparing experiments with relaxation cycles (consisting of 5 min of permeation and 1 min relaxation, with the same feed conditions but no permeation) and without relaxation cycles, under controlled conditions of permeate flux *J_perm_* of 18.8 L·h^−1^·m^−2^, at 40 °C and a Reynolds number at the feed compartment of 184. Table 3 shows the hydraulic permeability loss, the transmembrane pressure and the average permeability measured for each relaxation procedure.

**Figure 5:**
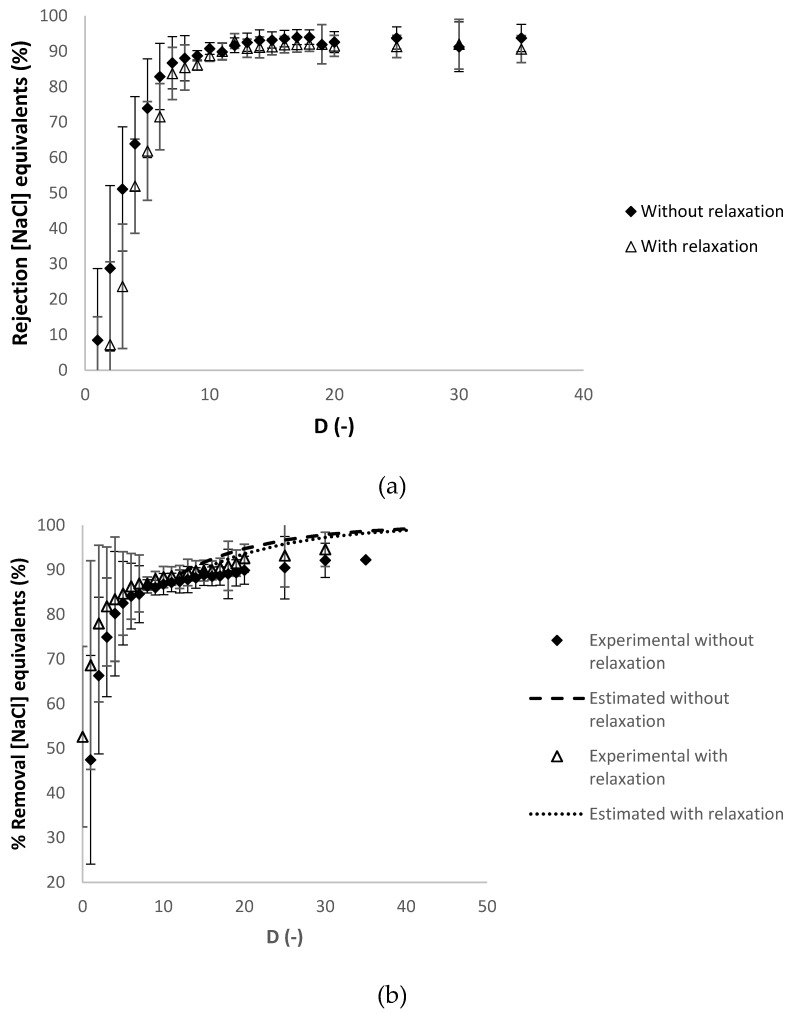
Dia-ultrafiltration with and without relaxation procedure with (diluted) arabinoxylans fraction as feed, at 40 °C, with a *Re_feed_* of 184: (**a**) $R_{NaCl}$ (% NaCl.eq), apparent rejection in sodium chloride equivalents and (**b**) percentage of removal of charged contaminants in sodium chloride equivalents (NaCl.eq) against the *D* (-) diafiltration volume. Each analysis had duplicate measurements.

As shown in Table 3, the experiment with relaxation cycles performed slightly better with slightly lower hydraulic permeability loss and transmembrane pressure and a slightly higher average permeability. Figure 5 presents respectively the progression of the apparent rejection factor (%) and the percentage of removal of the charged contaminants in NaCl equivalents against the diafiltration volume.

In Figure 5b, the percentage of removal of the charged contaminants was estimated without and with the relaxation cycles, at diafiltration volumes higher than 10, calculated with the experimental apparent rejection of charged contaminants at diafiltration volume of 10 (91% and 89%, respectively), which is considered constant in this range of diafiltration volumes (see Figure 5a).

**Figure 6:**
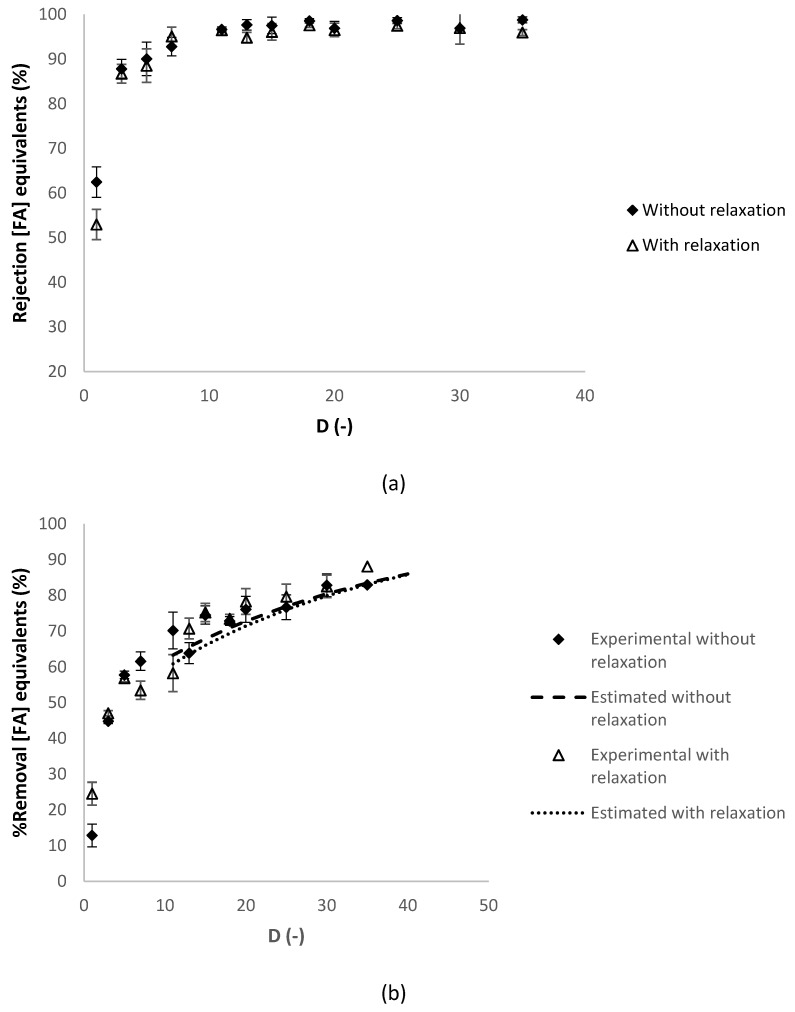
Dia-ultrafiltration with and without relaxation technique of membrane at 40 °C with a (diluted) arabinoxylans fraction as feed, at 40 °C, with an *Re_feed_* of 184: (**a**) $R_{FA}$ (%FA.eq), apparent rejection in ferulic acid equivalents and (**b**) percentage of removal of charged contaminants in ferulic acid equivalents (FA.eq) against the *D* (-) diafiltration volume. Each analysis had duplicate measurements.

A reasonable agreement between the experimental and the estimated values of the percentage of removal of the charged contaminants in sodium chloride equivalents is observed in both experiments. Figure 6 presents respectively the evolution of the apparent rejection factor (%) and the percentage of removal of the contaminants in ferulic acid equivalents against the diafiltration volume.

In Figure 6b, the percentage of removal of the contaminants was estimated without and with the relaxation cycles, at diafiltration volumes higher than 10, calculated with the experimental apparent rejection of contaminants at diafiltration volume of 10 (both at 97%), which is considered constant in this range of diafiltration volumes (see Figure 6a). A reasonable agreement between the experimental and the estimated values of the percentage of removal of the contaminants in sodium chloride equivalents is observed with and without the relaxation cycles. For the charged contaminants (in NaCl.eq), the apparent rejection of charged contaminants (see Figure 5a) and the percentage of removal (see Figure 5b) had similar values in both experiments. For the contaminants (in FA.eq), the apparent rejection (see Figure 6a) had similar results and the percentage of removal (see Figure 6b) had slightly higher values with relaxation cycles. In conclusion, the selected relaxation procedure was without relaxation cycles, as the relaxation cycles are more complex to operate and expensive and the benefits are not substantially better.

#### 3.2.3. Effect of the Reynolds Number at the Feed Compartment R_feed_

The impact of the Reynolds number at the feed compartment *R_feed_* (with defined values of 129 and 267) on the purification of the (diluted) raw material was evaluated by comparing experiments at 40 °C, without relaxation cycles, under controlled permeate flux conditions. Table 4 shows the hydraulic permeability loss, the transmembrane pressure, the volumetric flux and the average permeability measured for each value of Reynolds number at the feed compartment *R_feed_*.

In general, higher values of *R_feed_* mean better hydrodynamic conditions at the feed compartment, favoring better transport properties through the membrane and, thus, higher permeate fluxes J_perm_. The high increase of the *R_feed_* from 129 to 267 (around two times of the initial value) led to an increase of the hydraulic permeability loss and a significant increase in the transmembrane pressure *TMP* (around two times of the initial value), related to an increase of membrane fouling. The *TMP* is also the driving force of ultrafiltration membranes for the transport properties through the membrane, and this significant increase in the TMP also led to an increase in the permeate fluxes *J_perm_,* but a decrease in the average permeability (calculated as the ratio between the volumetric flux and the *TMP*).

**Figure 7:**
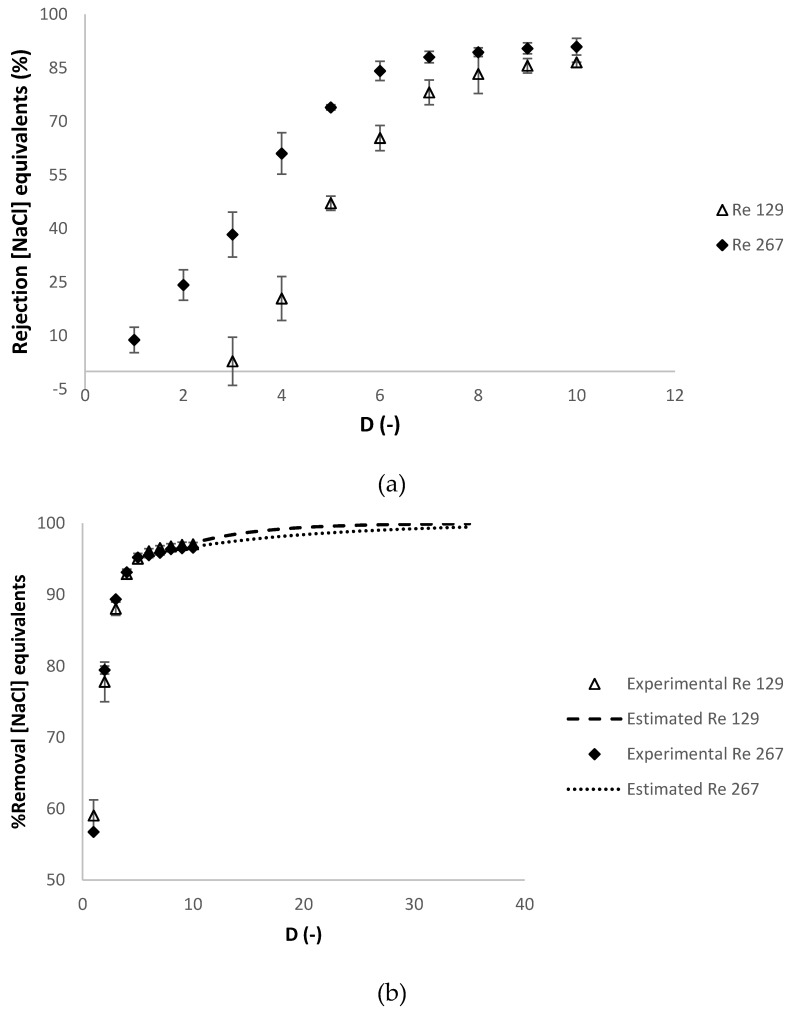
Dia-ultrafiltration at different Reynolds numbers at the feed compartment (129 and 267) experiments with (diluted) arabinoxylans fraction as feed, at 40 °C, without relaxation procedure: (**a**) *R_NaCl_* (% NaCl.eq), apparent rejection in sodium chloride equivalents and (**b**) percentage of removal of charged contaminants in sodium chloride equivalents (NaCl.eq) against the *D* (-) diafiltration volume. Each analysis had duplicate measurements.

The experiments were revised in this section, considering that in Section 3.2.1 and Section 3.2.2, 1—the values of apparent rejection of charged contaminants (in NaCl.eq) and of contaminants (in FA.eq) at diafiltration volume of 10 remained constant till the diafiltration volume of 35 and 2—there was a reasonable agreement between the experimental and estimated values of the percentage of removal of the charged contaminants (in NaCl.eq) and of the contaminants (in FA.eq). In order to have less time-consuming experiments in this section, the experiments were conducted until a final experimental value of diafiltration volume of 10. The values of the percentage of removal of the charged contaminants (in NaCl.eq) and of the contaminants (in FA.eq) were estimated until a final diafiltration volume of 35. Figure 7 presents respectively the progression of the apparent rejection factor (%) and the percentage of removal of the charged contaminants in NaCl equivalents against the diafiltration volume.

**Figure 8:**
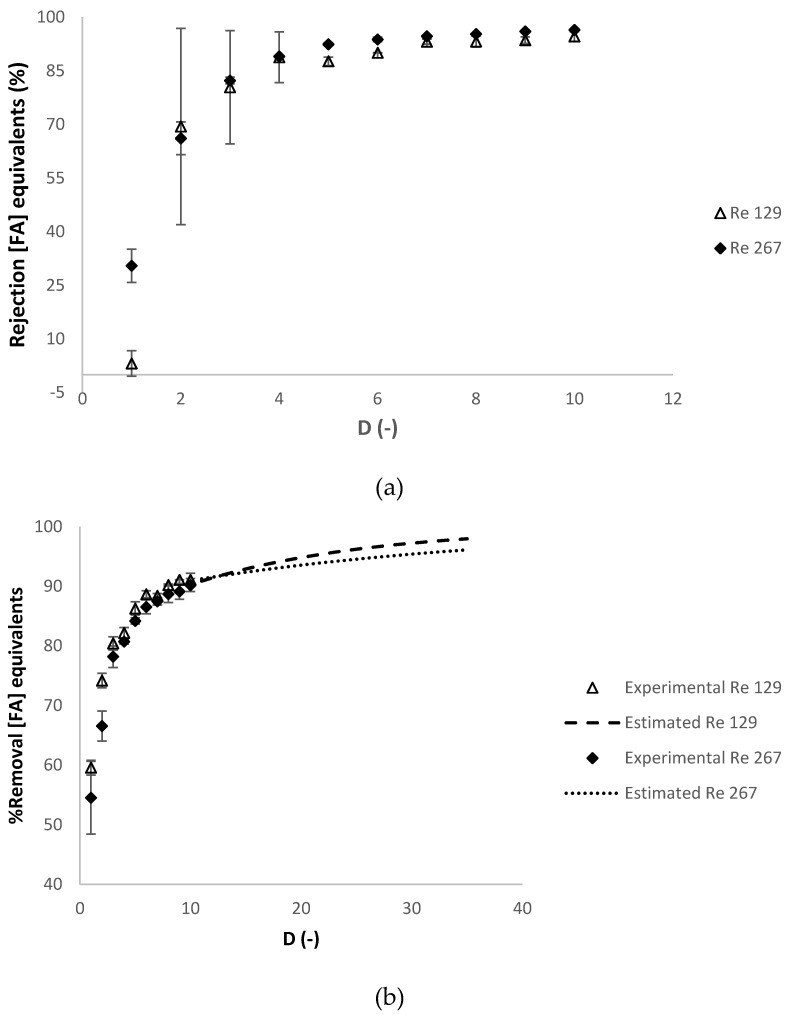
Dia-ultrafiltration at different Reynolds numbers (129 and 267); experiments with (diluted) arabinoxylans fraction as feed, at 40 °C, without relaxation procedure: (**a**) *R_FA_* (% FA.eq), apparent rejection in ferulic acid equivalents and (**b**) percentage of removal of charged contaminants in ferulic acid equivalents (FA.eq) against the *D* (-) diafiltration volume. Each analysis had duplicate measurements.

In Figure 7b, the percentage of removal of the charged contaminants was estimated at *R_feed_* of 129 and 267, at diafiltration volumes higher than 10, calculated with the experimental apparent rejection of charged contaminants at diafiltration volume of 10 (87% and 95%, respectively), which is considered constant in this range of diafiltration volumes (see Figure 7a). Figure 8a,b present the evolution of the apparent rejection factor (%) and the percentage of removal of the contaminants in ferulic acid equivalents against the diafiltration volume.

In Figure 8b, the percentage of removal of the contaminants was estimated at *R_feed_* of 129 and 267, at diafiltration volumes higher than 10, calculated with the experimental apparent rejection of contaminants at diafiltration volume of 10 (91% and 96%, respectively), which is considered constant in this range of diafiltration volumes (see Figure 8a).

The apparent rejection of charged contaminants (in NaCl.eq) and of the contaminants (in.FA eq) was slightly higher at the Reynolds number at the feed compartment of 267, but the percentage of removal of charged contaminants (in NaCl.eq) and the contaminants (in.FA eq) were similar. The selected Reynolds number at the feed compartment was 267, because, although it had higher membrane fouling effects, quantified by a higher value of hydraulic permeability loss and transmembrane pressure, it had higher volumetric permeate flux (turning the process more productive) and the quality of the final retentate obtained were similar in both experiments.

A rough comparison of two dia-filtration processes may be done (although various parameters differ), between the purification of an arabinoxylan fraction by dia-ultrafiltration (this work, with the purification selected operating conditions) and the purification of a pectin extract by dia-microfiltration [22]. In both processes, one type of contaminants content is quantified by the same measurement method (absorbances at 280 nm). At a diafiltration volume of 6, the percentage of removal of this type of contaminants was 87% in this work and 57% in [22]. Therefore, in this work, the process seemed to have performed better.

### 3.3. Films Characterization

#### Thickness, Color and Morphology

The films prepared were flexible when handled and presented a thickness of 0.10 ± 0.03 mm. The color parameters according to the CIEL*a*b* color space are shown in Table 5. The values obtained for both types of films (non-enriched and enriched in ferulic acid) are not significantly different (Tukey’s post-hoc test with a significance level of 0.05). Both films samples were translucid, presenting a yellowish color, which is agreement with the value calculated for the hue.

**Figure 9:**
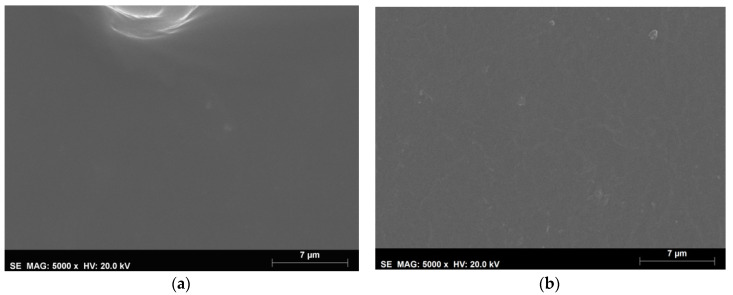
Microstructure of the arabinoxylan films: non enriched (**a**) and enriched with ferulic acid (**b**) obtained with the SEM technique at a magnification of 5000×.

The microstructure of the arabinoxylan films’ surface was observed using the scanning microscopy technique (Figure 9).

According to the images, for the magnification applied, the films prepared with arabinoxylans purified with the studied methodology present a continuous surface without visible pores or cracks. This morphology is consistent with that usually obtained for other polysaccharide films [30].

### 3.4. Water Vapor Permeability

Table 6 shows the water vapor permeability values of the films under study, along with that of arabinoxylan films from other authors, and from commercial cellophane and low density polyethylene (LDPE). The values for the films of the present work were significantly different (Tukey’s post-hoc test with a significance level of 0.05).

The high water vapor permeability value obtained for arabinoxylans’ films is attributed to their hydrophilic nature. Comparing the films of this study, we observe that the water vapor permeability increased when ferulic acid was incorporated in the film’s formulation. It is envisaged that ferulic acid may also act as plasticizer (in addition to glycerol) when its molecules are incorporated within the arabinoxylan polymeric matrix. These molecules may cancel some of the hydrogen bonds that are responsible to maintain the highly packed arabinoxylan molecules, eventually increasing intermolecular spaces and water vapor diffusion. In addition, the arabinoxylan films of this study present a lower permeability when compared to the other formulation shown in Table 6. Péroval et al. [28] produced arabinoxylan films from corn bran with 15% glycerol. A lower WVP value would be expected when using a lower plasticizer content, which was not the case. This fact may be attributed to several factors, namely to a different purity degree and average molecular weight of arabinoxylans and to the different methods used to prepare the films (e.g., drying conditions). The cellulose-based films of cellophane tested by the same authors show a permeability value in the same order of magnitude of that of arabinoxylan films without ferulic acid. Due to its hydrophobic nature, LDPE presents higher barrier properties to water vapor with a permeability value around 35 to 95 times lower than that of the hydrophilic films shown.

### 3.5. Mechanical Properties

#### Tensile Tests

The values of stress at break, deformation at break and Young modulus of the films of this study, along with that of arabinoxylan films from other authors, as well as from commercial cellophane and low-density polyethylene (LDPE), are shown in Table 7. The mechanical parameters values for the films of the present work were not significantly different (Tukey’s post-hoc test with a significance level of 0.05).

Regarding the films of this study, we may conclude that the amount of ferulic acid added did not affect substantially the mechanical properties under tensile tests. Compared to the films produced by Péroval et al. [28], similar stress and deformation at break values were observed, but a much higher Young modulus. This fact indicates that the films of the present study are more resistant to elastic deformation, even though they were produced with twice the amount of plasticizer (30% dry basis) when compared with the films of Péroval et al. (15% dry basis). As discussed above in the water vapor permeability section, the effect of different purity degree and average molecular weight of arabinoxylans and different methods used to prepare the films may have masked the effect of glycerol, resulting in polymeric matrices less resistant to elastic deformation, even with a lower content of plasticizer. All the hydrophilic films referred to in Table 7, though presenting a stress at a break value similar to that of LDPE, possess a much lower deformation at break. LPDE enables the production of quite ductile films (deformation at break from 100–965), which is a characteristic that is difficult to obtain for films from polysaccharides like arabinoxylans.

### 3.6. Films’ Antioxidant Activity

The antioxidant activity of the films prepared in this work was assessed with the FRAP method by direct immersion of a film sample in the reaction mixture. The values obtained were (1.56 ± 0.85) × 10^−5^ and (9.16 ± 1.50) × 10^−5^ mmol Trolox.mg^−1^ film, for non-enriched and enriched films with ferulic acid, respectively. Active films with increased antioxidant capacity were obtained by adding ferulic acid, a compound that is also present in the impurified arabinoxylans extract. There is the potential to design the purification methodology in order to obtain a purified extract with a designed ferulic acid concentration, enabling the production of active films with ferulic acid already present in the biomass instead of adding it in the pure form.

## 4. Conclusions

A detailed study was performed in this work on the purification of an arabinoxylan fraction (from corn fiber) by dia-ultrafiltration operated under controlled permeate flux conditions, with milder operating conditions than when operated under transmembrane pressure conditions. This study proved that this technology is suitable to purify, not only arabinoxylan fractions or extracts, but also other viscous streams, such as other polysaccharides extracts. Dia-ultrafiltration is also suitable to purify streams with compounds sensitive to shear stress, such as certain peptides or proteins. The purification by dia-ultrafiltration under permeate flux conditions (at 40 °C, with no relaxation procedure) allowed a removal of charged contaminants of 97% in NaCl equivalents and a removal of contaminants of 90% in ferulic acid equivalents at a final experimental value of diafiltration volume of 10. The percentage of removal of contaminants was able to be estimated at values of diafiltration higher than 10. The purified arabinoxylan extract was used successfully to produce arabinoxylan films with antioxidant activity. A good potential was envisaged of using this extract to design biodegradable films with suitable properties for industry applications, namely in the packaging of food products with low water content. The future goal of this work is to use this polysaccharide on the development of biodegradable films for food packaging (the main cause of sea pollution), which will be designed according to the different properties of foods and the wide range of environments. Arabinoxylans will be used, as well, for replacing the current synthetic polymers for membrane manufacture, since the disposal of the current non-sustainable materials represents an environmental burden.

## Figures and Tables

**Table 1:** Operating conditions studied during the purification of the (diluted) raw material (arabinoxylans fraction) by dia-ultrafiltration.

Operating Conditions Involved	Temperature (°C)	Relaxation Procedure ^1^	*Re_feed_* (-)
Temperature (°C)	30; 40; 50	40	40
Relaxation procedure	No relaxation	No relaxation, Relaxation cycles	No relaxation
Mode of operation	At *J_perm_* of 18.8 L·h^−1^·m^−2^	At *J_perm_* of 18.8 L·h^−1^·m^−2^	Under controlled *J_perm_* of 19.0 and 27.0 L·h^−1^·m^−2^
*Re_feed_* (-)	154; 184; 217	184	129; 267

^1^ The relaxation cycles comprised of 5 min of permeation and 1 min relaxation (same feed conditions but no permeation). Legend: *J_perm_*, is the permeate flux.

**Table 2:** Effect of the temperature on the purification of the (diluted) arabinoxylans fraction by dia-ultrafiltration operated without relaxation procedure and under controlled conditions of permeate flux *J_perm_* of 18.8 L·h^−1^·m^−2^: temperature *T* (°C); hydraulic permeability loss (%); transmembrane pressure *TMP* (bar); Reynolds number at the feed compartment and average permeability (L·h^−1^·m^−2^·bar^−1^).

Operating Conditions Imposed	Hydraulic Permeability Loss (%) ^1^	*TMP* (bar)	*Re_feed_* (-)	Average Permeability (L·h^−1^·m^−2^·bar^−1^)
T: 30 ± 2 °C	84 ± 1	0.44 ± 0.01	154	42.4 ± 0.2
T: 40 ± 2 °C	82 ± 2	0.46 ± 0.03	184	43.8 ± 0.1
T: 50 ± 4 °C	81 ± 5	0.41 ± 0.01	217	45.7 ± 0.4

^1^ during each purification experiment.

**Table 3:** Effect of relaxation on the purification of the (diluted) arabinoxylans fraction by dia-ultrafiltration operated under controlled conditions of permeate flux *J_perm_* of 18.8 L·h^−1^·m^−2^, at 40 °C and at a Reynolds number at the feed compartment of 184: hydraulic permeability loss (%); transmembrane pressure *TMP* (bar) and average permeability (L·h^−1^·m^−2^·bar^−1^).

Operating Conditions Imposed	Hydraulic Permeability Loss (%) ^1^	*TMP* (bar)	Average Permeability (L·h^−1^·m^−2^·bar)
Without relaxation	82 ± 2	0.46 ± 0.03	43.8 ± 0.1
With relaxation	73 ± 2	0.40 ± 0.03	52.7 ± 0.1

^1^ during each purification experiment.

**Table 4:** Effect of the Reynolds number at the feed compartment on the purification of the (diluted) arabinoxylans fraction by dia-ultrafiltration operated without relaxation procedure, under controlled conditions of permeate flux *J_perm_* of 19 L·h^−1^·m^−2^ and at 40 °C: hydraulic permeability loss (%); transmembrane pressure *TMP* (bar); flux (L·h^−1^·m^−2^); and average permeability (L·h^−1^·m^−2^·bar).

Operating Conditions Imposed	Hydraulic Permeability Loss (%) ^1^	*TMP* (bar)	*J_perm_* (L·h^−1^·m^−2^)	Average Permeability (L·h^−1^·m^−2^·bar)
Under controlled *J_perm_* and Re = 129	59 ± 12	0.31 ± 0.03	19.0	62.2 ± 0.4
Under controlled *J_perm_* and Re = 267	91 ± 3	0.60 ± 0.04	27.0	43.9 ± 2.8

^1^ during each purification experiment.

**Table 5:** Color experimental results for parameters a *, b *, L * and h.

Films	a *	b *	L *	h°
Arabinoxylans + 30% (*w*/*w* dry basis) glycerol	2.5 ± 1.05	42.54 ± 3.35	73.55 ± 2.98	86.64 ± 1.28
Arabinoxylan + 30% (*w*/*w* dry basis) glycerol + 10% (*w*/*w* solution) ferulic acid	1.78 ± 0.04	37.83 ± 0.51	76.27 ± 0.31	87.28 ± 0.04

**Table 6:** Water vapor permeability of arabinoxylan-based films and of commercial Cellophane and low density polyethylene (LDPE). WVP: water vapor permeability.

Film	Thickness (μm)	WVP (×10^−11^ mol·m·m^−2^ s^−1^ Pa)
Arabinoxylans + 30% (*w*/*w* dry basis) glycerol	90.0 ± 10.17	0.62 ± 0.02
Arabinoxylans + 30% (*w*/*w* dry basis) glycerol + 10% (*w*/*w* dry basis) ferulic acid equivalents	89.0 ± 35.81	2.06 ± 0.29
Arabinoxylans + 15% (*w*/*w* dry basis) glycerol [31]	90.8 ± 6.60	0.98 ± 0.03
Cellophane [31]	20	0.38 ± 0.04
LDPE [31]	25	0.01 ± 0.01

**Table 7:** Mechanical properties under tensile tests of arabinoxylan-based films and commercial Cellophane and LDPE.

Films	Thickness (μm)	Stress at Break (MPa)	Deformation at Break (%)	Young Modulus (MPa)
Arabinoxylans + 30% (*w*/*w* dry basis) glycerol	90.0 ± 10.2	21.7 ± 2.8	10.0 ± 1.0	274.5 ± 32.1
Arabinoxylans + 30% (*w*/*w* dry basis) glycerol + 10% (*w*/*w* dry basis) ferulic acid equivalents	89.0 ± 35.8	25.8 ± 3.1	17.0 ± 4.0	246.1 ± 35.2
Arabinoxylan + 15% (*w*/*w* dry basis) glycerol [31]	90.8 ± 6.6	26.5 ± 4.1	7.4 ± 2.9	72.4 ± 35.2
Cellophane [31]	20	114	20	-
LDPE [31,32]	25	13.1–27.6	100–965	-

## Supplementary Materials

The following are available online at https://www.mdpi.com/2077-0375/10/5/95/s1, Deduction of the equation to estimate the concentration of the retentate of contaminants present in this work.
