# Supplementary material for: Purification of Arabinoxylans from Corn Fiber and Preparation of Bioactive Films for Food Packaging
Source: Membranes (Basel). 2020 May 11;10(5):95. doi: 10.3390/membranes10050095 (PMC7281565; doi:10.3390/membranes10050095)
Supplement: Supplementary file 1 [file membranes-10-00095-s001.pdf]

membranes

MDPI

Supplementary Material

Purification of arabinoxylans from corn fiber and development of bioactive films for food packaging

Maria Serra [1], Verónica Weng [2], Isabel M. Coelhoso [2], Vitor D. Alves [1] and Carla Brazinha [2,*]

[1] LEAF—Linking Landscape, Environment, Agriculture and Food, Instituto Superior de Agronomia, Universidade de Lisboa, Tapada da Ajuda, 1349-017 Lisboa, Portugal; mariamanuelserra94@gmail.com (M.S.); vitoralves@isa.ulisboa.pt (V.D.A.)

[2] LAQV-REQUIMTE, Chemistry Department, Faculdade de Ciências e Tecnologia, Universidade Nova de Lisboa, 2829-516 Caparica, Portugal; v.weng@campus.fct.unl.pt (V.W.); imrc@fct.unl.pt (I.M.C.)

* Correspondence: c.brazinha@fct.unl.pt

Deduction of the equation to estimate the concentration of the retentate of contaminants present in this work

In this work, the purification of the (diluted) raw material was operated by ultrafiltration in a diafiltration mode (dia-ultrafiltration), where the concentration of the contaminants present (compounds i) in the feed compartment (the retentate) is estimated with a mass balance applied to the feed compartment, similar to a dia- nanofiltration process, but with important changes [1]:

$$\frac{d\left(V_{feed}\cdot c_{i,feed}\right)}{dt} = -J_v\cdot A \cdot c_{i,perm}$$

$$c_{i,feed}\cdot\frac{d\,V_{feed}}{dt} + V_{feed}\cdot\frac{d\,c_{i,feed}}{dt} = -J_v\cdot A \cdot c_{i,perm} \qquad \text{(S1)}$$

$$V_{feed}\cdot\frac{d\left(c_{i,feed}\right)}{dt} = -J_v\cdot A \cdot c_{i,perm}$$

where V_{feed} (L) is the volume of the feed compartment, *A* (m^2) is the membrane area and J_v ($L\cdot m^{-2}\cdot h^{-1}$) is the volumetric permeate and these parameters are constant during each experiment. The parameters $c_{i,feed}$ and $c_{i,perm}$ are respectively the retentate and the permeate concentrations of compounds *i* and vary along the permeation time, t. The value of permeation time t_1 (h) corresponds to when the apparent rejection of the contaminants *i*, R_i, becomes constant, till the end of the experiment. Consequently, Equation (S1) may be modified to (S1′)

$$V_{feed}\cdot\frac{d\left(c_{i,feed}\right)}{dt} = -J_v\cdot A \cdot c_{i,feed}\,.\,(1-Ri)$$

$$\int_{t_1}^{t} dt = -\frac{V_{feed}}{J_v\cdot A \cdot(1-R_i)}\int_{c_{i,feed}(t_1)}^{c_{i,feed}(t=t)}\frac{1}{c_{i,feed}}\,dc_{feed,i} \qquad \text{(S1)}'$$

The volume of permeate at a certain permeation time, V_P (L), is calculated by equations (S2) and (S3)

$$V_P = J_v\cdot A \cdot(t-t_1) \qquad \text{(S2)}$$

$$V_P = V_{feed} \cdot (D - D_1) \quad \text{(S3)}$$

where D (-) and D_1 (-) are the diafiltration volumes referred respectively to a certain permeation time t and to t_1 (h). In this case, equation (S1) is modified to (S1′′)

$$\int_{t_1}^{t} dt = -\frac{t-t_1}{(1-R_i)} \int_{c_{i,feed}(t_1)}^{c_{i,feed}(t=t)} \frac{1}{c_{i,feed}} dc_{feed,i}$$

$$-(D - D_1).\ (1 - R_i) = ln\left(\frac{c_{feed}(t)}{c_{feed}(t1)}\right) \quad \text{(S1′′)}$$

$$c_{i,feed}(t) = c_{i,feed}(t_1) \cdot exp[-(D - D_1) \cdot (1 - R_i)]$$

Reference

1. Almanasrah, M.; Brazinha, C.; Kallioinen, M.; Duarte, L.C.; Roseiro, L.B.; Bogel-Lukasik, R.; Carvalheiro, F.; Mänttäri, M.; Crespo, J.G. Nanofiltration and reverse osmosis as a platform for production of natural botanic extracts: The case study of carob by-products. *Sep. Purif. Technol.* **2015**, *149*, 389–397.
